# Evolving Strategies in Amblyopia Management: From Traditional Therapies to Cutting-Edge Innovations

**DOI:** 10.7759/cureus.88012

**Published:** 2025-07-15

**Authors:** Mina H Younan, Youssef T Youssef, Unaiza Erum, Muhammad Talal Nasir, Mohamed Ismaeil, Minar Aziz, Viola N Fawzy, Nadine Mourad, Mohamed Ismaiel, Mohamed Sherif Ali Ahmed, Lydia Melad, Momen Abdelglil

**Affiliations:** 1 Department of General Surgery, Hull University Teaching Hospitals NHS Trust, Hull, GBR; 2 Department of General Surgery, Mansoura University Faculty of Medicine, Mansoura, EGY; 3 Department of General Surgery, Foundation University Medical College, Islamabad, PAK; 4 Department of General Surgery, Royal Stoke University Hospital, Stoke-on-Trent, GBR; 5 Department of General Surgery, Prince Charles Hospital, Merthyr Tydfil, GBR; 6 Department of General Surgery, Aneurin Bevan University Health Board, Newport, GBR; 7 Department of Physiology, Suez Canal University, Ismailia, EGY; 8 Department of Ophthalmology, Alexandria University Hospitals, Alexandria, EGY; 9 Department of General Surgery, Royal Victoria Infirmary Hospital, Newcastle upon Tyne, GBR; 10 Department of Ophthalmology, Mansoura University, Mansoura, EGY; 11 Department of Ophthalmology, Beni-Suef University, Beni Suef, EGY; 12 Department of Pediatric Surgery, Mansoura University Children's Hospital, Mansoura, EGY

**Keywords:** amblyopia, neuroplasticity, ophthalmology, pediatric ophthalmology, vision therapy

## Abstract

Amblyopia, commonly referred to as "lazy eye," is a prevalent visual disorder affecting children and, in some cases, adults. Traditional treatments, including occlusion therapy and pharmacological approaches, have demonstrated efficacy but also face limitations related to adherence, age dependency, and recurrence rates. This narrative review explores evolving strategies in amblyopia management, from conventional therapies to emerging innovations. Dichoptic therapy, which leverages binocular visual stimulation, represents a paradigm shift by directly addressing neural suppression and improving stereopsis. Technological advancements, such as virtual reality (VR), AI, and eye-tracking technologies, have further revolutionized treatment approaches, enhancing compliance and enabling personalized care. Additionally, recent research in neuroplasticity has opened new avenues for treating adult amblyopia, challenging previous assumptions about its irreversibility. Despite these advancements, challenges persist in treatment standardization, accessibility, and long-term efficacy.

## Introduction and background

Amblyopia is a prevalent visual development disorder that affects approximately 1-5% of the global population. Traditionally, its management has relied on therapies such as refractive correction, occlusion therapy (patching), and pharmacological penalization. Although these conventional approaches have demonstrated efficacy in improving visual acuity in the amblyopic eye, they often face challenges, including limited compliance due to social stigma, discomfort, and psychological distress among patients and their families [1,2].

Emerging therapies, such as binocular dichoptic methods, virtual reality (VR)-based interventions, perceptual learning exercises, and non-invasive neuromodulation, aim to address not only monocular deficits but also binocular dysfunctions, including stereopsis. These novel approaches offer promising, more engaging, and patient-friendly alternatives, with improved adherence and favorable long-term outcomes [3]. This narrative review explores the evolution of amblyopia management strategies, from traditional therapies to cutting-edge innovations. By synthesizing recent evidence from clinical trials and systematic reviews, this article aims to provide a comprehensive overview of current practices while highlighting the potential of emerging technologies to improve the care of patients with amblyopia.

## Review

Understanding the condition of amblyopia

Amblyopia, or "lazy eye," is a common childhood vision disorder affecting 1% to 5% of children worldwide. It impairs visual acuity without visible eye abnormalities. The condition arises from disruptions in the brain’s visual pathways during development rather than structural defects. If left untreated, amblyopia can lead to severe and irreversible vision loss, emphasizing the importance of early detection and intervention [4-7]. Amblyopia develops due to visual deprivation, strabismus, or anisometropia. Strabismus disrupts binocular vision by causing eye misalignment, leading the brain to favor one eye while neglecting the other. Anisometropia creates a mismatch in visual perception due to differing refractive powers between the eyes. Visual deprivation, caused by conditions like ptosis or cataracts, obstructs vision in one eye and prevents proper sensory input. In all cases, the brain adapts by relying on the stronger eye, ultimately resulting in amblyopia. In the past, occlusion therapy, which involves patching the dominant eye to force the brain to utilize the weaker eye, was a major component of amblyopia treatment. Despite its effectiveness, this approach has drawbacks, such as poor adherence in children and limited success in older individuals. Furthermore, it primarily enhances visual acuity without adequately addressing deeper issues related to brain plasticity and binocular vision [8-12]. Modern amblyopia treatment is evolving with the use of interactive smartphone apps and VR tools that stimulate both eyes to reestablish binocular integration. Unlike traditional patching, these immersive techniques enhance visual sharpness and stereopsis by fostering coordinated visual input. Research indicates that these methods improve treatment compliance and may overcome some of the limitations of occlusion therapy [13-15]. In addition to digital technologies, pharmacological and neuromodulation therapies are also part of the future of amblyopia management. While atropine drops remain a conventional treatment, newer neuroenhancers show potential for increasing brain plasticity. Moreover, neuromodulation techniques such as transcranial direct current stimulation (tDCS) and transcranial magnetic stimulation (TMS) may enhance visual processing. The use of advanced neuroimaging to guide treatment is contributing to a shift toward more personalized and effective care for amblyopia [16-18].

Traditional management strategies for amblyopia

Early detection and intervention are essential, as treatment effectiveness decreases with age. Traditional amblyopia treatment focuses on balancing ocular dominance and achieving clear retinal images. This includes treating underlying conditions, prescribing corrective lenses, and using occlusion or penalization techniques on the dominant eye [19]. Patching has been a cornerstone of amblyopia treatment for over two centuries. It forces the weaker eye to develop by covering the stronger one. Research by the Pediatric Eye Disease Investigator Group (PEDIG) has shown that 6 hours of daily patching is as effective as full-time patching for severe amblyopia in children aged 3 to 7 years [16,19]. The duration of patching therapy has been extensively studied. For children with moderate amblyopia (20/40 to 20/80), 2 hours of occlusion therapy can initially yield improvements in visual acuity similar to those seen with 6 hours of patching. For severe amblyopia (20/100 to 20/400), two hours of daily patching results in an average improvement of 3.6 lines after 17 weeks. If no further improvement occurs, extending the therapy to 6 hours daily is recommended to enhance visual outcomes [16,20]. Another traditional approach is pharmacological penalization using atropine eye drops. This method involves applying a long-acting cycloplegic agent, typically 1% atropine sulfate, to the healthy eye to blur its vision and encourage use of the amblyopic eye. Studies have shown that daily atropine penalization can be as effective as patching for moderate amblyopia, with comparable improvements in visual acuity after six months of treatment [21,22]. While traditional treatments remain effective, advances in medical science and technology have introduced new possibilities. Home-based solutions, such as customized eye exercises and digital vision therapy apps, are gaining popularity. When combined with proper guidance and consistent effort, these methods can enhance traditional therapies and provide more accessible and empowering options for individuals affected by amblyopia. However, it is important to note that the effectiveness of these newer techniques is still under investigation and should be used under professional supervision [19,23].

Limitations of traditional therapies

Treatment Efficiency and Age Limitations

Recent evidence suggests that cortical plasticity may persist beyond the critical period (≈8 years), allowing amblyopia treatment to be effective in children of all ages, including older ones. A 2005 multicenter study by PEDIG found that 50% of treated children aged 7-12 showed significant improvement. Although the benefits were less clear in those over 12, some untreated cases in this group still responded to the intervention [24].

Limitations of Penalization Methods

Traditional treatments have primarily focused on penalizing the stronger eye using patching or pharmacological alternatives such as atropine. While these methods are effective to some extent, they face limitations related to patient comfort and overall effectiveness. Atropine penalization is easier to administer and is generally better accepted than patching; however, its potential toxicity and prolonged effects pose risks, particularly if reverse amblyopia occurs. Moreover, both atropine and patching result in similar visual improvements two years after treatment [25].

Pharmacological Treatments and Their Limitations

Side effects associated with levodopa are commonly reported in the literature and limit its clinical use. Reported adverse effects include nausea, headache, fatigue, mood changes, dizziness, dry mouth, decreased appetite, vomiting, and, in some cases, nightmares. However, further research, including a placebo-controlled trial suggested by the PEDIG group, is warranted to better evaluate its safety and efficacy [24].

Recurrence of Amblyopia

**Figure 1:**
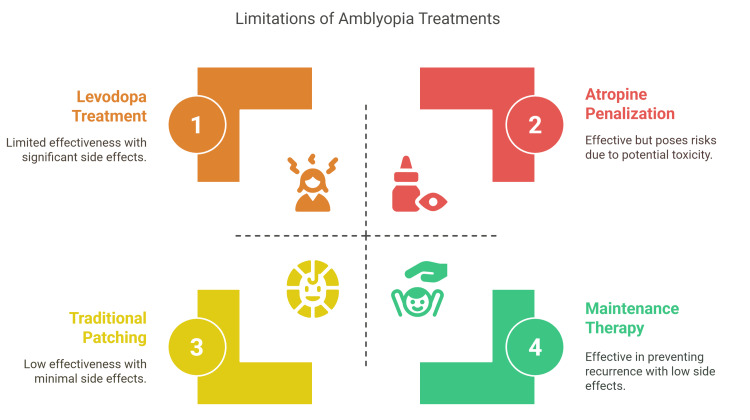
Limitations of traditional amblyopia therapy. Figure created by the article authors summarizing the limitations of traditional amblyopia treatments, including low effectiveness, side effects, and toxicity risks. While maintenance therapy helps prevent recurrence, it does not fully address initial visual deficits. Levodopa is noted for its limited efficacy and significant side effects, while atropine, though effective, carries toxicity risks. Traditional patching is associated with minimal side effects but also limited effectiveness. Overall, the figure communicates key therapeutic limitations in a clear, accessible, and visually engaging format. Source: References [24, 25].

A notable challenge in amblyopia management is the recurrence of symptoms following the cessation of therapy. Studies indicate that 13-24% of patients experience a decline in visual acuity within the first year after completing treatment. To reduce this risk, it is recommended that patients undergo a maintenance phase or gradual weaning from occlusion therapy (Figure 1) [24].

Dichoptic therapy: a new paradigm in amblyopia treatment

Dichoptic therapy represents a novel approach to amblyopia treatment that focuses on continuous stimulation of both eyes to reduce suppression and restore binocular vision. Traditional treatments like occlusion therapy often fail to address binocular function, whereas dichoptic therapy directly targets neural plasticity. By presenting distinct images to each eye, it promotes balanced input, thereby improving stereopsis and depth perception. Recent research highlights significant visual improvements in both children and adults undergoing this therapy. The potential of dichoptic therapy to replace or enhance traditional methods marks a crucial advancement in vision rehabilitation [26-30].

Mechanisms of Dichoptic Therapy

Dichoptic therapy relies on contrast-adjusted stimuli to enhance input from the amblyopic eye while maintaining balanced stimulation. This technique reduces interocular suppression and strengthens binocular summation. Adaptive training environments, including video games and VR platforms, further refine this process by engaging patients in visually stimulating tasks. Studies suggest that repeated exposure to dichoptic stimuli increases cortical response and facilitates long-term visual improvements. This mechanism is crucial for treating both childhood and adult amblyopia, ensuring sustained gains beyond the treatment period [26, 31, 32].

Technological Innovations in Dichoptic Therapy

Modern advancements have significantly enhanced the accessibility and effectiveness of dichoptic therapy. VR platforms create immersive environments that improve compliance and engagement, while augmented reality (AR) applications refine real-time visual interactions. AI contributes to adaptive treatment by tailoring exercises based on patient progress. These innovations ensure personalized treatment, making the therapy more effective and widely applicable. Ongoing research is exploring further refinements in VR-based interventions and AI-driven models to optimize treatment outcomes [33-35].

Limitations and Future Directions

Despite its advantages, dichoptic therapy faces several challenges, including patient variability, treatment compliance, and accessibility. Some individuals exhibit limited neuroplasticity, which can reduce treatment effectiveness. Inconsistent standardized protocols make it difficult to predict outcomes. Moreover, high-tech solutions such as VR-based therapies may not be accessible to all patients due to cost and availability. Addressing these barriers is essential to establish dichoptic therapy as a widely accepted and effective amblyopia intervention [24, 28, 35].

Future research should focus on refining treatment protocols and improving accessibility. Large-scale clinical trials are needed to develop universal guidelines for treatment duration and intensity. Combination therapies, such as pharmacological support to enhance neuroplasticity, are also being explored. Long-term follow-up studies will be crucial to assess the durability of treatment benefits. Advances in machine learning could further optimize therapy delivery, ensuring individualized care. Expanding these research avenues will help position dichoptic therapy as a mainstream solution for amblyopia and related visual disorders [4, 28, 33, 36].

Advances in digital technologies for amblyopia management

Amblyopia has traditionally been treated using methods such as patching and corrective lenses. However, with advances in technology, the condition is now benefiting from digital innovations that have significantly improved treatment outcomes and patient satisfaction. Numerous mobile apps are available to treat amblyopia through interactive exercises and visual tasks. These are designed to encourage the lazy eye to engage in visual activity by interacting with images or games [19,37].

VR devices are being used to treat amblyopia by engaging both eyes in visually demanding tasks that require binocular attention. These VR-based treatments offer customized exercises to improve eye coordination and depth perception, making them a key tool in amblyopia therapy. Mobile apps specifically designed for amblyopia treatment help make therapy more engaging, particularly for children. Recently, video games have been developed that require visual tasks stimulating the weaker eye, turning therapy into an enjoyable experience. One such example is the "Amblyopia Treatment via Computer" (AT-300). Smart glasses are another example of wearable devices that aid in amblyopia treatment by either stimulating both eyes or encouraging use of the weaker eye. These may employ adaptive optics, block vision in the dominant eye, or use filter-based treatments [38-40]. Devices like the Occlusion Therapy System use patches or glasses to occlude the dominant eye and can be digitally controlled for precise adjustments. These systems also provide feedback to both patients and clinicians regarding treatment progress [41].

Advanced Eye Tracking Technology

Eye tracking systems are increasingly used to evaluate the effectiveness of amblyopia therapies, especially in dichoptic treatments. In a multicenter randomized clinical trial, an eye-tracking-based home therapy was employed to guide real-time adjustments in visual stimuli, ensuring accurate binocular engagement. This technology not only monitored the coordination between both eyes but also allowed therapy to be personalized and adapted based on patient performance, resulting in improved treatment adherence and outcomes [42].

AI

AI algorithms analyze data from eye tests and digital screenings to develop personalized treatment regimens. These systems can track progress, adjust interventions based on patient response, and identify subtle visual patterns to aid in early and accurate diagnosis. AI tools such as Visulytix and EyeArt have shown promise in enhancing detection and decision-making in ophthalmic care, while platforms like Optos AI support widefield retinal imaging interpretation. These technologies enable individualized amblyopia treatment planning and monitoring, especially in regions with limited access to specialists [19].

Telemedicine for Early Diagnosis and Remote Monitoring

Advancements in telemedicine and portable technologies have made remote screening for amblyopia increasingly feasible. Smartphone-based apps, such as GoCheck Kids, and software like Photo Vision Screener (PVS) enable parents and primary care providers to conduct preliminary vision assessments at home or in remote clinics. These tools utilize photo-screening technology to detect risk factors for amblyopia and are particularly beneficial in underserved or rural areas with limited access to pediatric ophthalmologists. Additionally, virtual consultations through secure digital platforms allow for remote monitoring of patient progress and adjustment of treatment plans as needed [43-45].

Neuroplasticity and Adult Amblyopia: New Opportunities

Neuroplasticity refers to the brain's ability to reorganize itself by forming new neural connections throughout life, allowing for learning, adaptation, and recovery from injury. Amblyopia, or "lazy eye," arises when one eye experiences reduced vision due to the brain favoring the other, leading to weakened visual pathways and long-term impairment. Recent advancements in our understanding of neuroplasticity reveal that the brain can restructure its visual pathways, offering new possibilities for treating adult amblyopia, a condition once thought to be untreatable [32,46].

tDCS is a non-invasive technique that uses low electrical currents applied to the scalp to stimulate specific brain regions, such as the visual cortex. This stimulation enhances the brain's ability to process visual information and promotes neuroplasticity. When combined with traditional therapies like patching or visual training exercises, tDCS may improve visual acuity in adults with amblyopia by accelerating the rewiring of neural pathways [47,48].

Perceptual learning is another promising approach that involves repetitive practice of challenging visual tasks to improve function in the amblyopic eye. This method encourages the brain to adapt and form new connections to compensate for the reduced input from the weaker eye. By engaging both eyes, perceptual learning strengthens the brain’s binocular processing abilities, improving overall vision [46].

**Figure 2:**
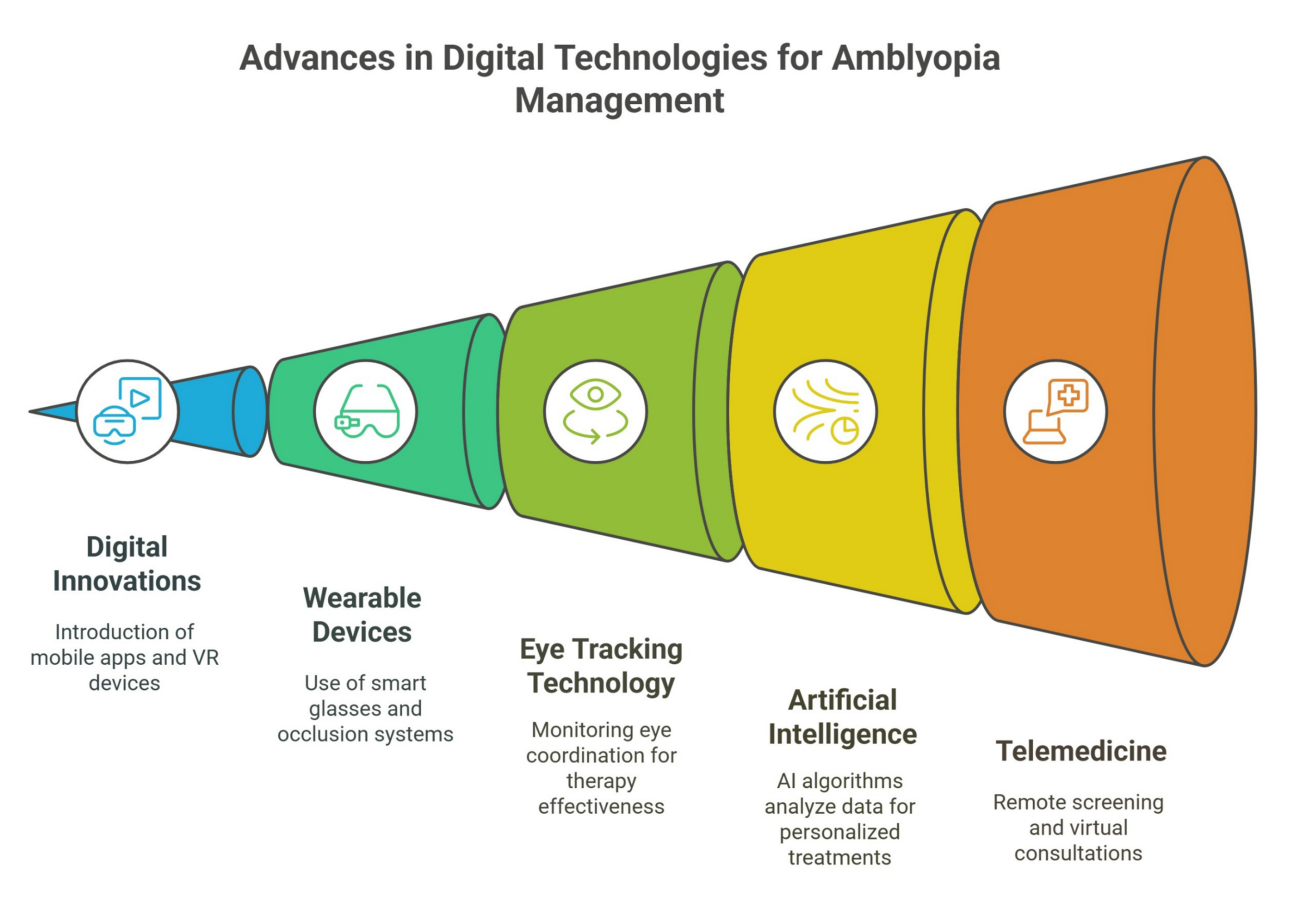
Advances in digital technologies for amblyopia management. This infographic illustrates the progressive integration of digital innovations in amblyopia care. It begins with mobile applications and virtual reality (VR) tools designed to engage patients, followed by wearable devices such as smart glasses and digital occlusion systems. Eye-tracking technology supports real-time monitoring of ocular coordination, while AI enables the creation of personalized treatment plans. The pathway concludes with telemedicine, which facilitates remote screening and virtual consultations, particularly beneficial for patients in underserved regions. Sources: References [37-48].

The growing understanding of neuroplasticity has significantly enhanced amblyopia treatment in adults, especially when combined with tDCS, perceptual learning, and immersive technologies such as VR and AR. These innovative approaches are showing promising results in overcoming the limitations of traditional therapies. Ongoing research continues to refine these methods, offering new hope for adults with amblyopia and challenging the long-held belief that the condition is untreatable (Figure 2) [32,47].

Challenges and future directions in amblyopia research

Amblyopia continues to face challenges in both diagnosis and treatment. One major concern is the recurrence of visual acuity decline following cessation of therapy. Studies have shown that 13-24% of patients experience regression within the first year after stopping treatment, particularly if therapy is discontinued abruptly or without a maintenance phase. Recurrence is more likely in younger children, and in cases where initial improvement was rapid or substantial, without adequate stabilization time [19].

Progress in screening technologies is expected to strengthen amblyopia research and improve early detection. Traditional screening methods, such as visual acuity charts and photoscreeners, have shown false positive rates ranging from 21% to 50%, particularly in preschool-aged children, which has led to skepticism among healthcare providers [19, 43]. In contrast, AI-based screening tools and binocular retinal birefringence scanning have demonstrated higher specificity and lower false positive rates, making them promising alternatives for early diagnosis. However, these AI tools also carry potential risks, such as bias in training data, lack of algorithm transparency, and reduced generalizability across diverse populations. Ongoing research is needed to validate these technologies in broader clinical settings [19, 43-45].

Emerging solutions, including AI-based screenings and retinal birefringence assessments, offer more accurate risk evaluation and enable timely interventions, especially in underserved populations. However, inconsistent implementation across healthcare systems underscores the need for further research and policy development [19, 37].

Addressing social determinants of health is also crucial in amblyopia research. Children from underprivileged or unclear backgrounds may benefit less from standard treatments due to barriers such as low awareness, limited access, and difficulty maintaining therapy adherence. Future studies should aim to identify these challenges and develop targeted interventions to enhance accessibility and effectiveness. Engaging parents through educational sessions may improve adherence and treatment outcomes. Additionally, a comprehensive classification system incorporating genetic and epigenetic markers could help predict treatment responses and enable personalized care approaches. The integration of novel therapies with traditional methods may further improve visual outcomes and quality of life for affected individuals [23].

Despite promising advances in digital and AI-driven amblyopia therapies, several critical challenges remain. Many of these technologies lack long-term data on treatment durability and sustainability. Furthermore, younger children or those with cognitive impairments may encounter usability issues with interactive platforms. Cost and infrastructure barriers also persist in rural and low-income settings, potentially widening the accessibility gap. Additionally, home-based digital therapies often require close parental supervision, which may not be feasible for all families. Finally, concerns over excessive screen time in pediatric patients call for careful application and further research to ensure a balance between therapeutic benefits and developmental well-being.

## Conclusions

Amblyopia requires early intervention to prevent permanent vision loss. Although traditional treatments like occlusion therapy and atropine remain the standard of care, they are limited by issues of adherence and variable efficacy. Newer approaches, such as dichoptic therapy, digital training, neuromodulation, and AI-driven methods, show promise in improving compliance and enhancing binocular function. Future research may explore the integration of wearable technology, AI-based dosing algorithms, and home-based VR platforms to personalize therapy and improve access in underserved regions. Combining AI, VR, and real-time telemonitoring could ultimately enable adaptive, clinician-guided care at scale, transforming amblyopia treatment into a globally accessible solution. Advances in neuroplasticity have also expanded treatment possibilities beyond childhood. However, challenges in accessibility, treatment standardization, and long-term outcome validation persist, underscoring the need for continued research and technological integration to further enhance amblyopia care.
